# Cavin-3 (PRKCDBP) deficiency reduces the density of caveolae in smooth muscle

**DOI:** 10.1007/s00441-017-2587-y

**Published:** 2017-03-11

**Authors:** Baoyi Zhu, Karl Swärd, Mari Ekman, Bengt Uvelius, Catarina Rippe

**Affiliations:** Department of Experimental Medical Science, Lund University, BMC D12, 223 84 Lund, Sweden

**Keywords:** Caveolin-1, Cavin-1, Vascular function, Detrusor

## Abstract

Cavins belong to a family of proteins that contribute to the formation of caveolae, which are membrane organelles with functional roles in muscle and fat. Here, we investigate the effect of cavin-3 ablation on vascular and urinary bladder structure and function. Arteries and urinary bladders from mice lacking cavin-3 (knockout: KO) and from controls (wild type: WT) were examined. Our studies revealed that the loss of cavin-3 resulted in ∼40% reduction of the caveolae protein cavin-1 in vascular and bladder smooth muscle. Electron microscopy demonstrated that the loss of cavin-3 was accompanied by a reduction of caveolae abundance by 40-45% in smooth muscle, whereas the density of caveolae in endothelial cells was unchanged. Vascular contraction in response to an α_1_-adrenergic agonist was normal but nitric-oxide-dependent relaxation was enhanced, in parallel with an increased relaxation on direct activation of soluble guanylyl cyclase (sGC). This was associated with an elevated expression of sGC, although blood pressure was similar in WT and KO mice. Contraction of the urinary bladder was not affected by the loss of cavin-3. The proteomic response to outlet obstruction, including STAT3 phosphorylation, the induction of synthetic markers and the repression of contractile markers were identical in WT and KO mice, the only exception being a curtailed induction of the Golgi protein GM130. Loss of cavin-3 thus reduces the number of caveolae in smooth muscle and partly destabilizes cavin-1 but the functional consequences are modest and include an elevated vascular sensitivity to nitric oxide and slightly disturbed Golgi homeostasis in situations of severe cellular stress.

## Introduction

Many cell types, such as vascular smooth muscle cells and endothelial cells, are abundantly endowed with small plasma membrane invaginations termed caveolae. These structures, which are rich in cholesterol and sphingolipids, might act as signaling platforms for receptors that convey messages from the surrounding environment into the cell (Parton and del Pozo 2013). Caveolae are generated by proteins that are inserted into the inner leaflet of the cell membrane. These proteins are called caveolins (Cav-1 to Cav-3) and associate with cytosolic proteins called cavins (cavin-1 to cavin-4). The latter proteins form a complex that aid in the formation of caveolae by providing a stabilizing cytoplasmic coat. Caveolin-1 was the first caveolae protein to be discovered and was identified as being necessary for caveolae to form. The cavins were discovered more recently and their requirement for the formation of caveolae is cell-type-dependent. Deficiency in either caveolin-1 or cavin-1 results in the complete absence of caveolae, together with the breakdown of other caveolae proteins. The role of cavin-2, -3 and -4 has been less well investigated but a recent study demonstrated that a lack of cavin-2 results in the loss of endothelial caveolae in the lung (but not in the heart), whereas the loss of cavin-3 has no effect on endothelial or fibroblast caveolae (Hansen et al. 2013; Liu et al. 2014). Another study indicated that the knockdown of cavin-3 reduces caveolae by 87% in a human fibroblast cell line (Hernandez et al. 2013). These findings reinforce the view that the less well-studied cavins, namely cavin-2 and -3, contribute to caveolae in a cell- or species-specific manner, although an in vivo role for cavin-3 in the formation of caveolae has yet to be definitively established.

Cavin-3 was originally identified as a protein that binds to protein kinase C-δ and hence, its gene name is *PRKCDBP* for Protein Kinase C Delta Binding Protein. The stability of caveolins and cavins has been found to depend on the presence of the caveolar protein complex and caveolins and cavins are targeted for degradation in the absence of this stabilizing structure. Accordingly, cavin-3 expression depends on cavin-1 and on caveolin-1 but not on cavin-2 (Hansen et al. 2013). Studies have shown that cavins form trimers that can be composed either of three cavin-1 molecules or of a mix of two cavin-1 molecules with one cavin-2 or one cavin-3 molecule (Ludwig et al. 2013; Kovtun et al. 2014). These trimers are then used as building blocks in higher-order assembly (Stoeber et al. 2016). Because cavin-1 and cavin-2 can substitute for cavin-3 in such trimers, the lack of cavin-3 might be compensated for by other cavins that are present at adequate concentrations. A reasonable starting point in the search of a role for cavin-3 for caveolae formation in vivo should thus be to identify tissues expressing high levels of cavin-3 compared with other cavins.

Here, we sought to establish the role of cavin-3 in caveolae formation in vivo. To this end, we surveyed cavin-3 expression and found it to be preferentially expressed in smooth muscle. In keeping with this tissue distribution, we found that caveolae were less abundant in vascular and urinary bladder smooth muscle cells from cavin-3-deficient mice. The physiological consequences of cavin-3 deficiency were mild, however, involving increased vascular relaxation in response to a nitric oxide (NO) donor and slightly higher expression of soluble guanylyl cyclase (sGC). Growth and protein expression in the urinary bladder in response to outlet obstruction, a model of distension-induced growth, were nevertheless largely unchanged. These studies establish a role of cavin-3 in the formation of caveolae in vivo but also show that the physiological consequences of cavin-3 ablation are modest.

## Materials and methods

### Animals

Mice, namely *Prkcdbp* knockout (KO) and wild-type (WT) on a B6.12954 background, were purchased from the Jackson Laboratory and were propagated (KO × KO, WT × WT) and housed in the animal facility at BMC in Lund, Sweden. Both KO and WT mice bred well and no dramatic difference in litter size was apparent. Mice were kept on a 12/12 h dark/light cycle and had free access to food and water. Animals were used at an age between 10–20 weeks. The experimental protocols were approved by the local Malmö/Lund Ethics Committee (M433-12 and M46-13) at Lund University and all efforts were made to minimize suffering.

### Transmission electron microscopy

Small mesenteric arteries were fixed by immersing the tissue in 2.5% glutaraldehyde in 0.1 M sodium cacodylate buffer (pH 7.4). Urinary bladders were excised from perfusion-fixed animals. Briefly, each mouse was anesthetized by isoflurane (4%) and the thoracic cavity was opened. A small incision was made in the left ventricle, a catheter was advanced into the aorta and another incision was made in the right atrium. After washes with phosphate-buffered saline (PBS), fixative was infused into the mouse and the bladder was excised. The tissues were post-fixed in 1% osmium tetroxide for 2 h, stained with uranyl acetate, dehydrated and embedded in Araldite. Sections were first stained with toluidine blue to choose areas to be cut for electron microscopy. Images of endothelial cells and the underlying smooth muscle cells were taken at 60 K magnification in a JEOL JEM 1230 microscope (Jeol, Tokyo, Japan). Caveolae were quantified by tracing the cell membrane in the images and counting the vesicles with a size of 50–100 nm that resided within 100 nm from the cell membrane by using ImageJ (NIH, Bethesda, Md., USA).

### Immunohistochemistry

Small mesenteric arteries, caudal arteries and urinary bladders were fixed in 4% paraformaldehyde and embedded in paraffin. Sections (5 μm thick) were cut, mounted on slides and deparaffinized. Sections were incubated overnight (4 °C) with primary antibodies dissolved in PBS (pH 7.2) containing 3% bovine serum albumin and 0.25% Triton X-100. The primary antibody used was rabbit anti-cavin-3 (16250-1-AP, ProteinTech group, Chicago, Ill., USA) and sections were subsequently rinsed (2 × 10 min) in PBS with 0.25% Triton X-100 followed by incubation with secondary antibody. For fluorescence imaging, secondary antibodies with specificity for rabbit and coupled to Cy2 (Jackson, West Grove, Pa., USA) were used and nuclei were stained with 4,6-diamidino-2-phenylindole (DAPI). Fluorescence micrographs were captured via an Olympus DP72 microscope equipped with a digital camera and by using Olympus CellSensDimension software. For immunohistochemistry of the bladder, the tissue was blocked with 4% H_2_O_2_ in methanol for 20 min at room temperature to inhibit endogenous peroxidase activity. Visualization of cavin-3 expression was performed by using secondary antibodies conjugated with HRP (horseradish peroxidase; Cell Signaling) at a 1:200 dilution and nuclei were counterstained with hematoxylin (Histolab).

### Western blotting

Arteries and bladders were homogenized in 70 μl SDS-buffer (62.5 mM TRIS–HCl pH 6.8, 2% SDS (w/v), 10% (v/v) glycerol, protease inhibitor, phosphatase inhibitor) by using a Tissue Lyser (Qiagen) as described (Turczynska et al. 2012). Samples (5-20 μg) were separated on Criterion TGX Precast Gels (Bio-Rad) and proteins were transferred to nitrocellulose membranes by using the Trans-Blot Turbo system (Bio-Rad). Visualization of proteins of interest was performed by subjecting the membrane to primary antibodies (caveolin-1, Cell Signaling; cavin-1, cavin-2 and cavin-3, Abcam; GAPDH, Millipore; GUCY1A3, Thermo Scientific; GUCY1B3, Proteintech). Appropriate secondary antibodies (Cell Signaling) were employed and finally, the membranes were developed by using SuperSignal West Femto Substrate (Thermo Scientific). The resulting chemiluminescence was analyzed by using Fc Odyssey (Li-Cor) and Image Scope software.

### Vascular reactivity

The tail artery or the small mesenteric arteries were dissected in Ca^2+^-free HEPES (135.5 mM NaCl, 5.9 mM KCl, 1.2 mM MgCl_2_, 2.5 mM CaCl_2_, 11.6 mM glucose, 11.6 mM HEPES, pH 7.4), mounted in wire myographs (DMT) and stretched to 5 mN (Sward et al. 2014). All vessels were contracted twice at 25 min intervals with 60 mM K^+^ HEPES, before the protocol was started. Tail arteries were contracted with incremental doses of cirazoline (3 × 10^−9^-10^−6^ M) and relaxed by using incremental concentrations of carbachol (10^−8^-10^−4^ M) in the presence or absence of L-NAME (L-N^G^-nitroarginine methyl ester; 3 × 10^−7^ M). After contraction with 10^−6^M cirazoline, relaxation in response to SNP (sodium nitroprusside; 10^−8^-10^−5^ M) and the cGMP activator BAY 41–2272 (3 × 10^−9^- 3 × 10^−6^ M) was investigated in tail arteries. Small mesenteric arteries were subjected to the vasoconstrictors serotonin (2 × 10^−6^ M) or angiotensin II (Ang-II; 3 × 10^−6^ M). Small mesenteric arteries were also mounted in a pressure myograph system in Ca^2+^-free solution containing 2 mM EGTA. Passive vessel diameter was measured in response to incremental elevation in pressure (45–120 mmHg).

### Blood pressure measurements

Blood pressure was measured in conscious mice by using a tail-cuff system (CODA, Kent Scientific) as described (Krawczyk et al. 2015). Mice (WT and KO) were adapted to the holders and the cuffs on three different occasions, separated by at least 3 days, before the actual blood pressure measurement.

### Partial bladder outlet obstruction

Mice were subjected to partial bladder outlet obstruction as described (Shakirova et al. 2010). Briefly, mice were anesthetized by using isoflurane (2%) and outlet obstruction was achieved by tying 6–0 Prolene around the urethra and a 0.4-mm stainless steel spacer via an abdominal incision. Following the placing of the Prolene, the spacer was removed, creating a standardized partial obstruction. Sham-operated mice, undergoing the same procedure but without tightening of the ligature, were used as controls. Mice were killed after 7 days by using increasing CO_2_ and bladders for biochemical analyses were rapidly excised, emptied and immersed in liquid N_2_. Bladder lysates were then prepared as described above.

#### Statistics

Means ± SEM are depicted in the figures. Single comparisons were made by using a two-tailed Student’s *t*-test. Concentration response curves were compared by using RAMNOVAs and multiple comparisons were made by using ANOVAs followed by Bonferroni post-hoc tests. RNA-Seq data from the GTExPortal was downloaded and TMM-normalized as described (Krawczyk et al. 2015). All analyses were made by using GraphPad Prism; *n* denotes the number of animals. *P* < 0.05 was considered as significant (*P < 0.05, **P < 0.01 and ***P < 0.001).

## Results

### Cavin-3 deficiency results in reduced number of caveolae in vascular smooth muscle cells

**Fig. 1:**
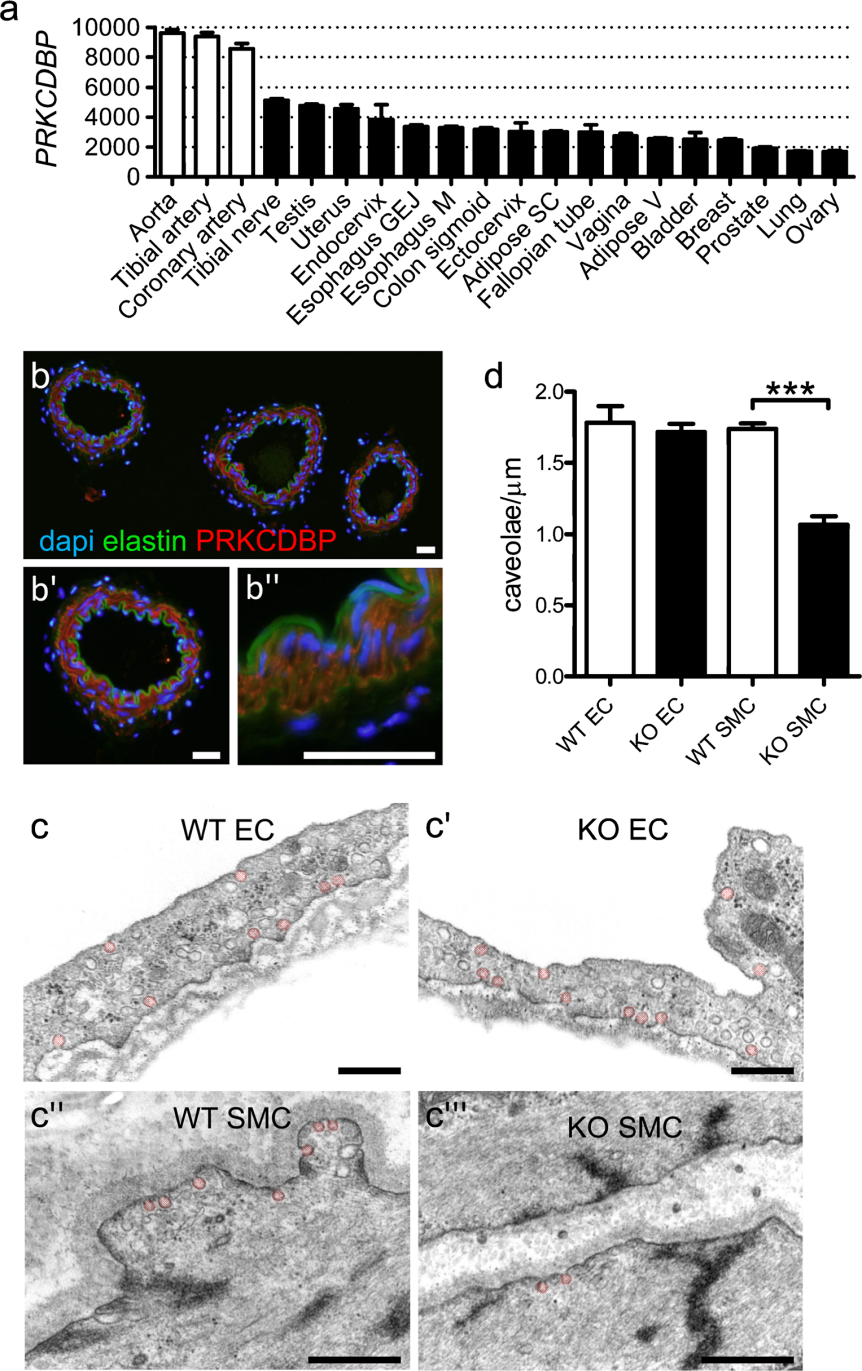
Cavin-3 (*PRKCDBP* protein kinase C delta binding protein) is highly expressed in arterial smooth muscle cells and contributes to the formation of caveolae. To identify tissues expressing high levels of cavin-3 (*PRKCDBP*), we analyzed mRNA expression data from GTExPortal.org (**a**), showing preferential expression in arteries (*white bars*). **b–b’’** Small mesenteric and caudal arteries from mice were subjected to immunofluorescence staining by using an antibody against cavin-3 (*red*). Nuclei were stained with DAPI (*blue*) and elastic laminae were visualized by using autofluorescence (*green*). Cavin-3 appeared specific for the medial smooth muscle cells as shown in the images at higher magnification (**b’**, **b’’**). *Bars* 25 μm. **c** Representative electron micrographs of small mesenteric arteries from wild-type (*WT*) and cavin-3 knockout (*KO*) mice. **c**, **c’** Caveolae in endothelial cells (*EC*). **c’’**, **c’’’** Caveolae in smooth muscle cells (*SMC*). Selected caveolae are highlighted in *pink*. *Bars* 500 nm. **d** Number of caveolae per micrometer plasma membrane in endothelial and smooth muscle cells analyzed in three mice per genotype and covering total membrane lengths of 485 and 479 μm in WT and KO, respectively. ***P < 0.001

To explore the tissue distribution of cavin-3, we used RNA-Seq data from the GTEx Portal (http://www.gtexportal.org/). We found that human arteries (aorta, tibial artery and coronary artery) expressed the highest levels of cavin-3 mRNA among all tissues represented in the database (Fig. 1a shows the top 20 expressing tissues). Because arteries contain various cell populations (smooth muscle, endothelial and adventitial cells), we next stained mouse arteries to determine in which cells cavin-3 was expressed. We found the medial smooth muscle layer between the elastic laminae (green, Fig. 1b) to be positive for cavin-3 (red). No staining of endothelial cells inside the internal elastic lamina was apparent. Adventitial cells were similarly negative. We next compared the membrane density of caveolae in endothelial and smooth muscle cells in small mesenteric arteries from WT and KO mice by using electron microscopy (Fig. 1c). The density of caveolae in endothelial cells was similar in WT and KO mice (Fig. 1c’ vs. c’’, d), whereas the caveolae density in smooth muscle cells was reduced by ≈ 40% (Fig. 1c’’ vs. c’’’, d). We concluded that cavin-3 contributed to the biogenesis of caveolae in vascular smooth muscle cells in vivo.

### Loss of cavin-3 reduces expression of cavin-1 in arteries

**Fig. 2:**
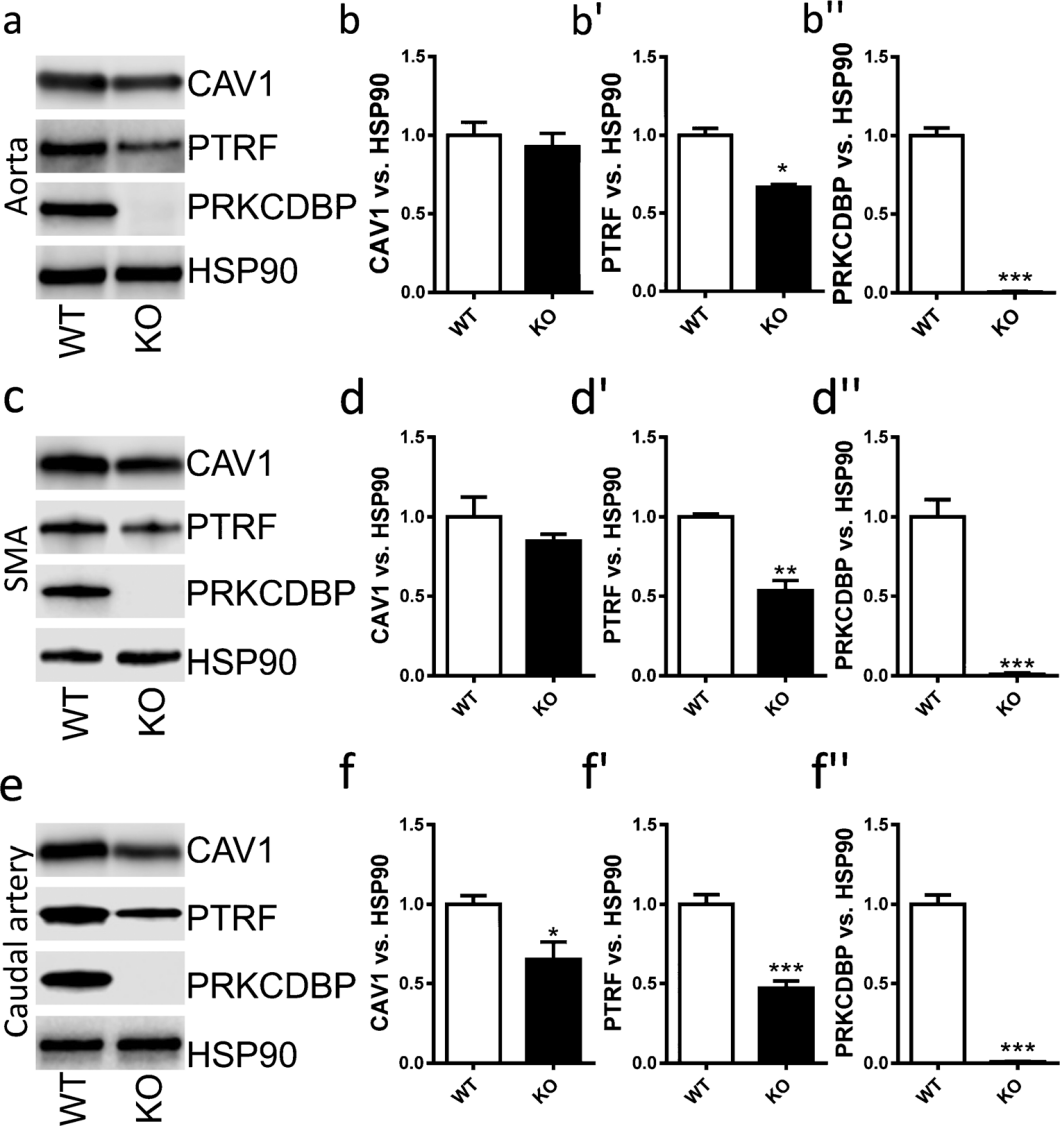
Destabilization of cavin-1 (PTRF) in the vascular wall in cavin-3-deficient mice. Protein expression was determined by Western blotting in aorta (**a**, **b-b’’**; *n* = 9 per group), in small mesenteric arteries (**c**, **d-d’’**; *n* = 3 per group) and in caudal artery (**e**, **f-f’’**; *n* = 5 WT, *n* = 6 KO) from WT and KO mice. Protein (5 or 20 μg) was separated on SDS-polyacrylamide gels and transferred to nitrocellulose membranes that were incubated with antibodies recognizing the various caveolae-associated proteins, namely caveolin-1 (CAV1), cavin-1 (PTRF) and cavin-3 (PRKCDBP). Heat-senstive protein 90 (HSP90) was used as a loading control. *P < 0.05, **P < 0.01 and ***P < 0.001

Knock-down of either caveolin-1 or cavin-1 typically results in reduced levels of other caveolae-associated proteins. We therefore next explored whether the loss of cavin-3 had any effect on the expression of other caveolae proteins in three different arteries (aorta, small mesenteric artery, caudal artery). We found that cavin-1 (polymerase I transcription and release factor, PTRF) was significantly reduced in all of these arteries (Fig. 2a-f). Caveolin-1 similarly tended to be reduced throughout but this difference reached significance only in the caudal artery (Fig. 2e, f), which is a comparatively muscular artery. We concluded that the lack of cavin-3 was associated with reduced levels of cavin-1 at the tissue level.

### Increased sensitivity to NO in cavin-3 KO mice

**Fig. 3:**
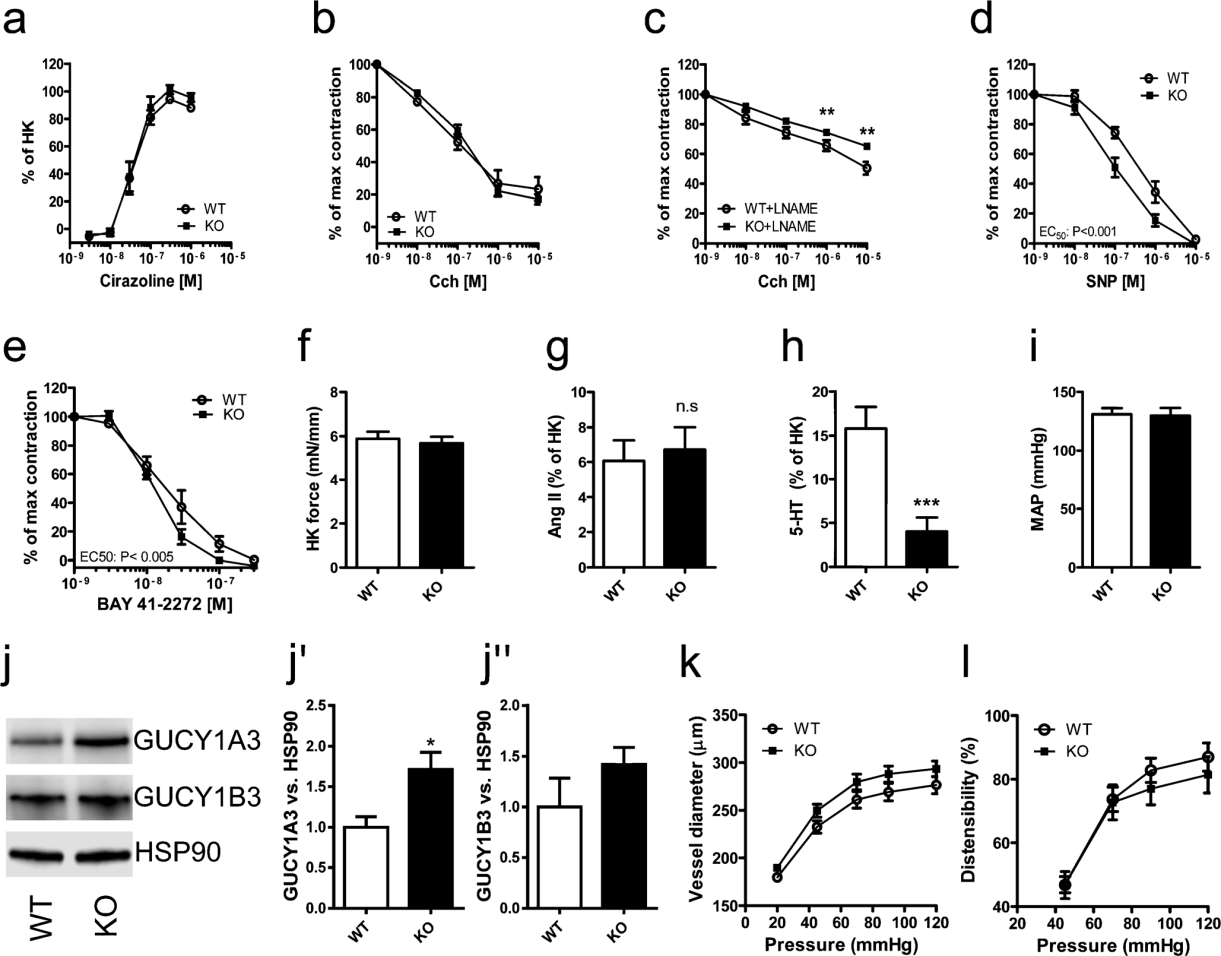
Nitric-oxide-dependent component of dilation is increased and L-NAME-resistant component of dilation is reduced in absence of cavin-3. Arteries from WT and cavin-3-deficient (KO) mice were dissected and mounted in wire-myographs for force recordings. Contraction was induced in caudal arteries by using incremental doses of the α_1_-adrenergic agonist cirazoline (*n* = 9; *HK* high-potassium; **a**). After a maximal contraction in response to cirazoline, relaxation to incremental doses of acetylcholine was assessed in the absence and presence of the nitric oxide synthase inhibitor L-NAME (*Cch* carbachol; **b**, **c**). Arteries were also relaxed by cumulatively increasing concentrations of sodium nitroprusside (*SNP*; *n* = 9; **d**) and the soluble guanylyl cyclase (sGC) activator BAY 41–2272 (*n* = 6; **e**). Contraction in response to high-potassium (*HK*, 60 mM K in HEPES) was assessed before the start of the experiments (**f**). Contraction in response to angiotensin II (*AngII*) and 5-HT (*n *= 9 per group) was assessed in the small mesenteric artery (**g**, **h**). Systemic blood pressure (*MAP* mean arterial pressure) was assessed in conscious mice by using a tail-cuff system (**i**). Expression of the two sGC subunits Gucy1a3 and Gucy1b3 was measured by Western blotting (*HSP90* heat-sensitive protein 90; **j-j’’**). Small mesenteric arteries were also dissected and mounted in a pressure myograph under calcium-free conditions. Pressure was increased incrementally, vessel diameter was measured and distensibility was calculated (**k**, **l**). *P < 0.05, **P < 0.01, ***P < 0.001, *n.s.* not significant

Vascular smooth muscle cells contract and regulate arterial diameter and this is considered to depend on caveolae. We therefore mounted arteries from WT and KO mice in wire and pressure myographs to assess arterial function directly. Contraction in response to the α_1_-adrenergic agonist cirazoline was similar in WT and cavin-3 KO arteries (Fig. 3a), as was total relaxation in response to the muscarinic agonist carbachol (Fig. 3b). In the presence of the NO synthase inhibitor L-NAME, pre-contracted KO vessels showed a reduced ability to relax in response to charbachol (Fig. 3c), suggesting a larger nitric-oxide-dependent component (i.e., relaxation in the absence of L-NAME minus relaxation in the presence of L-NAME). This was further assessed by investigating relaxation in response to an NO-donor (SNP) and a guanylyl cyclase activator (BAY 41–2272). We found that cavin-3 KO arteries were significantly more sensitive to SNP and to BAY 41–2272 (Fig. 3d, e) compared with WT arteries. Contraction in response to depolarization and angiotensin II was unaffected by the lack of cavin-3 (Fig. 3f, g), whereas contraction in response to 5-HT was reduced (Fig. 3h). To assess whether these changes in vascular function had any physiological consequences, we measured the blood pressure of conscious mice; no difference in blood pressure between WT and KO mice was detectable (Fig. 3i). To examine further the basis of the enhanced relaxation by SNP and BAY 41–2272, both of which activate sGC in smooth muscle cells, we determined the expression of Gucy1a3, one of the subunits of sGC and found it to be increased (Fig. 3j, j’). Gucy1b3 tended also to be increased but this was not significant (Fig. 3j’’). Measurements of passive vessel characteristics in the pressure myograph revealed no clear-cut differences in either vessel diameter or in distensibility (Fig. 3k, l). Taken together, these experiments showed that the lack of cavin-3 was associated with increased relaxation in response to the activation of sGC in vitro but that in vivo, this was compensated for such that overall endothelial-dependent relaxation and blood pressure remained similar. Some, but not all, responses to agonists at G-protein-coupled receptors were moreover reduced.

### Cavin-3^−/−^ mice have reduced amount of caveolae in bladder smooth muscle but show no change during muscarinic contraction

**Fig. 4:**
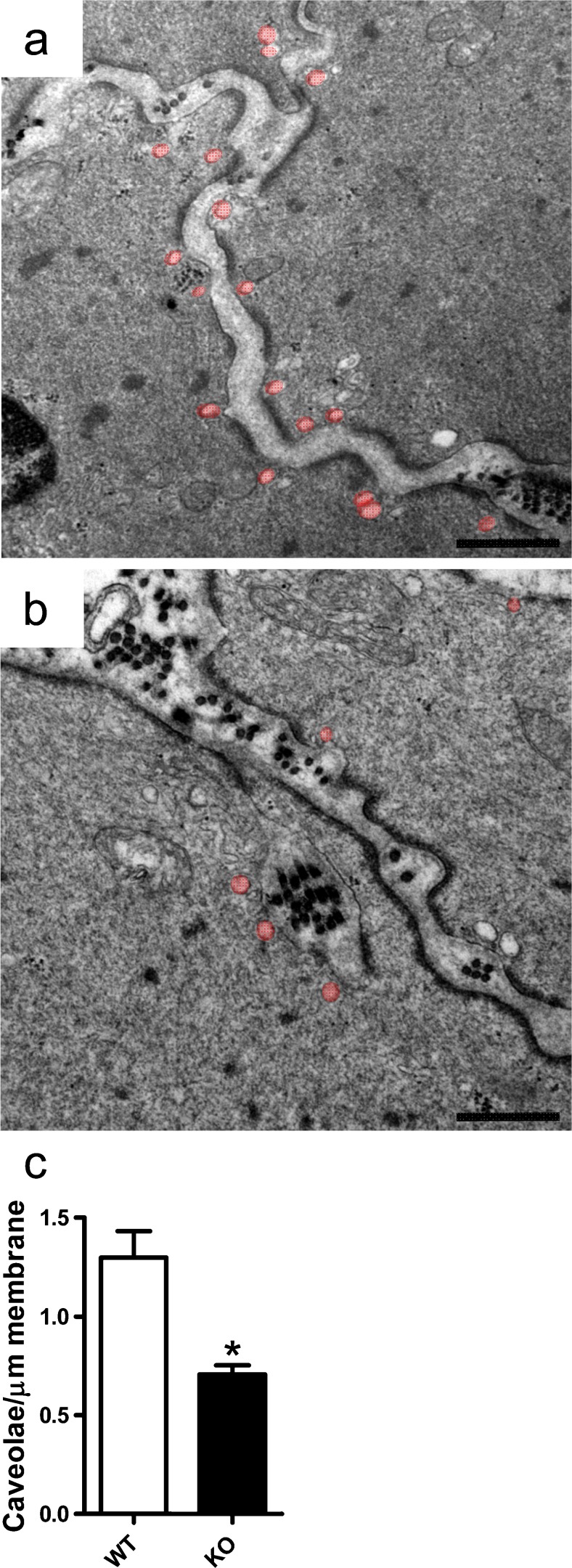
Reduced density of caveolae in bladder smooth muscle from cavin-3 knockout mice. Following perfusion fixation, urinary bladders from wild-type (*WT*) and cavin-3-deficient (*KO*) mice were excised and prepared for electron microscopy. **a**, **b** Electron micrographs of detrusor myocytes from WT (**a**) and KO (**b**) mice. Caveolae are highlighted in *pink*. *Bars* 500 nm. **c** Number of caveolae per micrometer plasma membrane in bladder smooth muscle cells from three mice per genotype and covering total membrane lengths of 583 and 614 μm in WT and KO, respectively. *P < 0.05

**Fig. 5:**
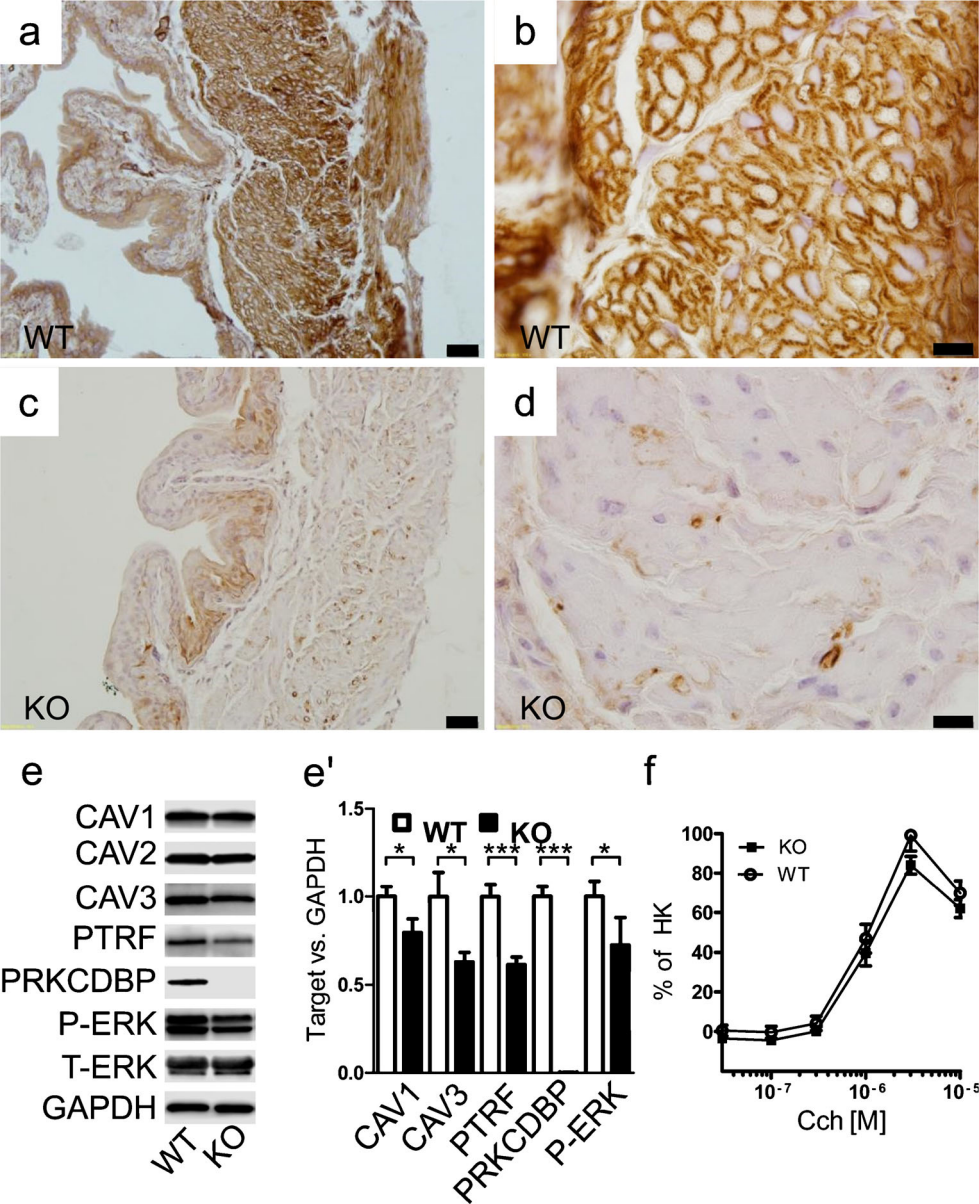
Cavin-3 is expressed in detrusor myocytes and its absence is associated with reduced expression of caveolin-1, caveolin-3 and cavin-1 but leaves contractility unchanged. Bladders from wild-type (*WT*) and cavin-3-deficient (*KO*) mice were processed for immunohistochemistry with cavin-3 antibody (*n* = 3 per group; **a–d**). Cavin-3 (*brown*) was mostly localized to smooth muscle cell membranes, as shown in the high-magnification cross-section in **b** and was absent from this location in KO mice (**d**). Caveolins, cavins, phosphorylated extracellular signal-regulated kinase (P-ERK) and ERK were determined by Western blotting (**e**, **e’**; *n* = 13 per group). Contraction of bladder strips following treatment with increasing concentrations of carbachol was measured (**f**; *n* = 6 per group). Force was normalized to depolarization-induced contraction (HK) but similar results were obtained when absolute force was plotted (not depicted). *P < 0.05, ***P < 0.001

To assess whether the reduction of caveolae density in cavin-3-deficient mice applied to other smooth muscles, we examined the urinary bladder. We found that cavin-3 KO mice had approximately 45% fewer caveolae in bladder smooth muscle cells compared with WT mice (Fig. 4a-c). Staining for cavin-3 in WT mice was intense in membranes of the smooth muscle layer but this was completely absent in KO mice (Fig. 5a–d). Some staining in the mucosa and of a rare cell type in the muscle layer remained in KO mice, suggesting that the antibody bound to additional but less abundant, epitopes under some conditions. The expression of caveolin-1, caveolin-3 and cavin-1 was significantly reduced in KO bladder, as shown by Western blotting (Fig. 5e). We also measured total extracellular signal-regulated kinase (T-ERK) and phosphorylated ERK (P-ERK) and found a slight decrease in P-ERK in KO mice (Fig. 5e, e’). To assess whether the reduction of caveolae had contractile consequences, we measured force in response to stimulation with the muscarinic agonist carbachol but found no significant difference between genotypes (Fig. 5f). We concluded that the lack of cavin-3 was also associated with a reduction of caveolae in urinary bladder smooth muscle cells.

### Cavin-3 deficiency does not affect bladder growth but diminishes induction of Golgi protein GM130 in response to partial bladder outlet obstruction

**Fig. 6:**
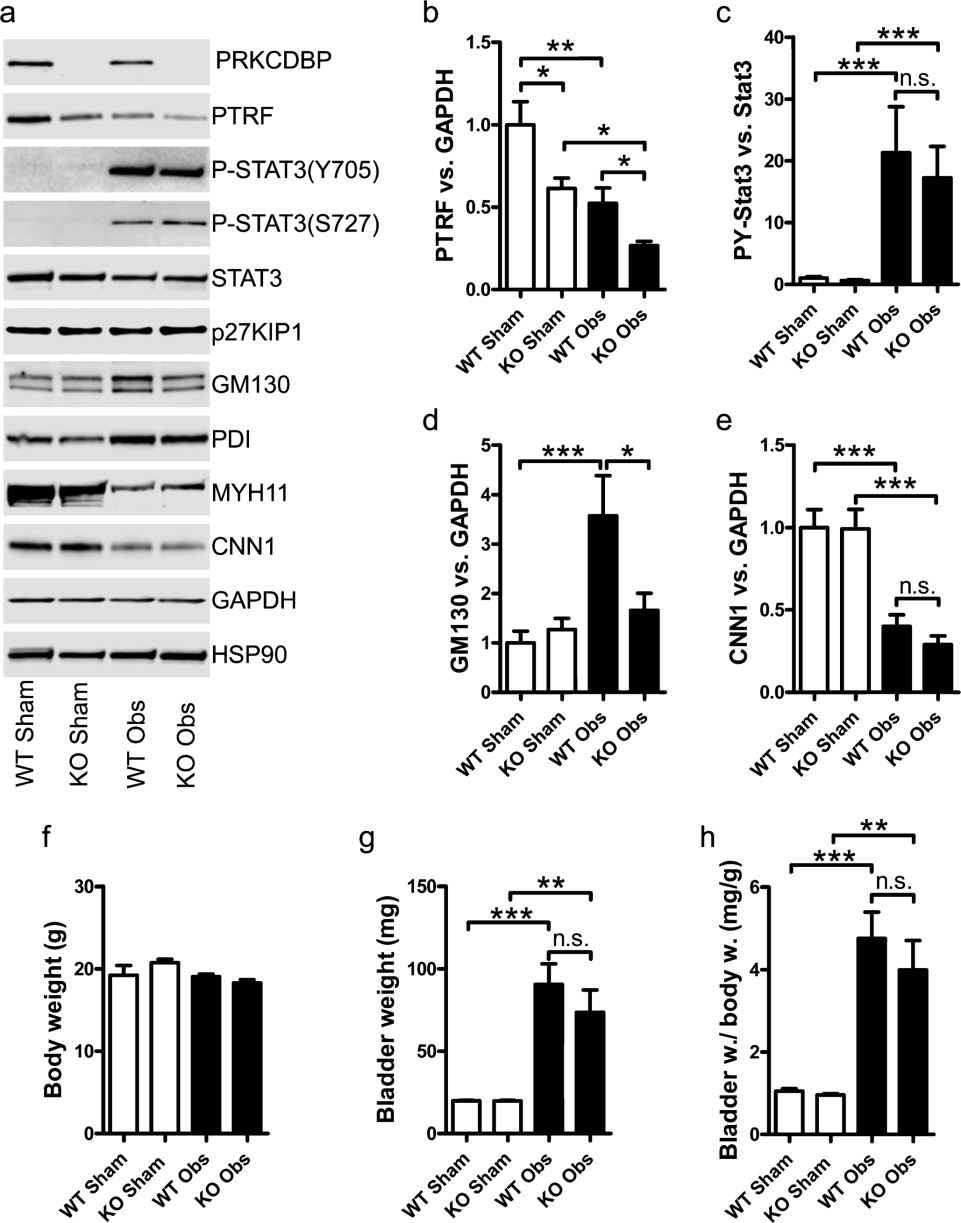
Preserved bladder growth and proteomic response to outlet obstruction in the absence of cavin-3. WT and KO mice were sham-operated or subjected to partial bladder outlet obstruction (*n* = 6 per group). After 7 days, bladders were harvested and the specified proteins were determined by Western blotting (**a**). Some of the proteomic changes, including changes in PTRF, PY-STAT3, GM130 and calponin, are depicted as bar graphs in **b–e**. **f**, **g** Body weights and bladder weights in control and obstructed WT and KO mice. **h** Bladder to body weight ratio. *P < 0.05, **P < 0.01, ***P < 0.001, *n.s.* not significant

Since the lack of cavin-3 did not affect detrusor function, we decided to stress the system by inducing partial bladder outlet obstruction. Bladder outlet obstruction is an in vivo model (Shakirova et al. 2010) that induces sizeable bladder growth via mechanical distension of the bladder and that associates with defined proteomic changes, including STAT3 activation (Fujita et al. 2006), induction of the endoplasmic reticulum (ER)-stress markers PDI (protein disulfide isomerase) and GM130 and repression of contractile markers such as myosin heavy chain (MYH11) and calponin (CNN1; Krawczyk et al. 2016). Cavin-1 (PTRF) was reduced by bladder outlet obstruction and, in keeping with our previous results, cavin-1 was lower in both sham-operated and obstructed KO mice (Fig. 6a, b). The majority of the remaining changes studied were unaffected in the absence of cavin-3: phosphorylation of the pro-proliferative transcription factor STAT3 and induction of the ER-chaperone PDI were unchanged (Fig. 6a, c), as was the expression of the cell cycle inhibitor p27KIP (CDKN1B, Fig. 6a) and repression of the differentiation markers MYH11 and CNN1 (Fig. 6a, e). The only difference was that the Golgi-associated protein GM130 was less forcefully induced by obstruction in the absence of cavin-3 (Fig. 6a, d). In keeping with an unchanged proteomic response to obstruction, we found that bladder growth (Fig. 6f-h) was equivalent in WT and KO mice. Thus, despite a marginally lower basal phosphorylation of ERK and impeded induction of GM130, the overall growth response of the urinary bladder following outlet obstruction remained intact.

## Discussion

The current study shows that cavin-3 (PRKCDBP) contributes to the formation of caveolae in smooth muscle in vivo. Cavin-3 ablation reduced the density of caveolae in smooth muscle cells by 40-45%, whereas caveolae abundance was maintained in endothelial cells. This difference in dependence on cavin-3 is probably attributable to the negligible expression of cavin-3 in endothelial cells, which instead express cavin-2, albeit in an organ-specific manner (Hansen et al. 2013). A recent model has suggested that cavins form a net-like lattice that constitutes the cytoplasmic coat of caveolae (Stoeber et al. 2016). Whether the size of the proposed polyhedral building blocks of this coat is affected by the cavin composition is unknown but we note that a difference in the size of caveolae in endothelial and smooth muscle cells has been reported (Sward et al. 2014), with a somewhat smaller size distribution of caveolae in smooth muscle compared with in the endothelium. This difference might tentatively be dictated by the repertoire of cavins expressed in the various cell types (cavin-1 and -2 or cavin-1 and -3) but numerous other factors such as membrane lipid composition and caveolin isoform expression might also contribute.

A consistent finding in our study was that the lack of cavin-3 was associated with a reduced level of cavin-1 (PTRF). This was seen in three different arteries and in the urinary bladder. A less consistent and smaller, reduction of caveolin-1 was also seen. Normally, caveolins have a slow turnover rate but when caveolae formation is disturbed, the rate of degradation is accelerated. For example, early studies on caveolin-1 KO mice demonstrated that caveolin-2 was redistributed, possibly to the Golgi apparatus and degraded (Razani et al. 2001; Drab et al. 2001). One degradative path of caveolae proteins involves targeting to late endosomes and lysosomes but evidence also suggests the involvement of the proteasome pathway (Razani et al. 2001; Hayer et al. 2010). The dependence of caveolins and cavins on higher-order protein assemblies is further emphasized by the finding that the ablation of cavin-1 destabilizes all caveolins and cavin-2 (Liu and Pilch 2008; Bastiani et al. 2009). Whether cavin-3 is degraded by ablation of caveolae might be cell-type-dependent and the knockdown of cavin-1 has been found to destabilize cavin-3 in 3T3-L1 cells but hardly has any effect on heart and liver (Bastiani et al. 2009). Our own work on cavin-1-deficient urinary bladders (Karbalaei et al. 2012) and small mesenteric arteries (Sward et al. 2014) has demonstrated the reduction of cavin-3, together with all caveolins. From these observations, we infer that cavin-3 contributes to higher-order protein assemblies in some cells but not in others and that this is dictated, at least in part, by its tissue concentration.

A robust finding in our prior studies on small arteries from caveolin-1 and cavin-1 KO mice was the increased response to the α_1_-adrenergic agonist cirazoline, which scaled with an increased wall thickness (Shakirova et al. 2006; Albinsson et al. 2007; Sward et al. 2014). This was not seen in the present work, which also did not reveal a change in arterial dimensions or distensibility, possibly because of the partial loss of caveolae in contrast with the complete, or almost complete, loss of caveolae in caveolin-1 and cavin-1 KOs. Alternatively, the medial remodeling and increased α_1_-adrenergic responsiveness reported in caveolin-1 and cavin-1 KOs might be indirect and mediated by the loss of caveolae in endothelial cells. Tissue-specific deletion of caveolin-1 or cavin-1 is required to distinguish between these possibilities.

Our experiments suggest an increased NO-dependent component of relaxation in cavin-3-deficient small arteries. Numerous prior studies have provided evidence for the activation of endothelial NO synthase (eNOS) in caveolin-1 KOs (Razani et al. 2001; Zhao et al. 2002; Desjardins et al. 2008) and in cavin-1 KOs (Sward et al. 2014), an effect that is considered to depend on the loss of an inhibitory interaction between caveolin-1 and eNOS in the endothelium (Kraehling et al. 2016). Changes at the protein level of Ddah1 and Arg1, which indirectly impact on eNOS activity, in cavin-1 KOs are expected partly to compensate for this effect (Sward et al. 2013). Against this background, we were surprised to find an increased NO-dependent component of relaxation that was associated with increased sensitivity to SNP and to Bay 41–2272 in a model that uniquely targets caveolae in smooth muscle. Reported changes of SNP-induced relaxation in caveolin-1-deficient animals do not seem to be directionally consistent and both increased (Pojoga et al. 2014) and decreased (El-Yazbi et al. 2005) responses have been reported. We cannot currently explain these results but we should emphasize that our findings in cavin-3 KO arteries are internally consistent, with increased relaxation going hand in hand with the increased expression of one of the subunits of the sGC. Of further interest is that L-NAME-resistant relaxation to carbachol, which is considered to depend on endothelial-dependent hyperpolarization (EDH; Figueroa and Duling 2009), is reduced. This is in good agreement with the reduction of EDH seen in global caveolin-1 KO mice (Saliez et al. 2008) and argues that a defined fraction of the caveolae-dependence of the EDH mechanism can be ascribed to smooth muscle cells. Clearly, however, all functional changes that we find at the resistance artery level are inconsequential for mean arterial blood pressure, which is well maintained. This is consistent with the majority of prior studies in caveolin-1-deficient mice (Rahman and Sward 2009).

A reduced density of membrane caveolae in cavin-3-KO mice was not unique for arterial smooth muscle but was also seen in the urinary bladder. The comparatively large biomass of the bladder allowed a somewhat more detailed proteomic characterization, showing a reduction of caveolin-3 in addition to caveolin-1 and cavin-1. We also considered a number of proteins recently shown to change after the knockdown of cavin-3 in fibroblasts (Hernandez et al. 2013) but among these, only phosphorylated ERK was reduced. This suggests adaptive mechanisms in live animals. We did not detect any change in contractility of bladder strips in vitro contrasting with reported contractile deficits in both caveolin-1-deficient (Woodman et al. 2004; Sadegh et al. 2011) and cavin-1-deficient (Karbalaei et al. 2012) detrusors. Because caveolin-1 staining in the bladder is remarkably specific for the smooth muscle, with no staining of the urothelium and only faint staining of the submucosa (Sadegh et al. 2011), we propose that the unchanged bladder contractility in cavin-3-KO animals must be attributable to the incomplete ablation of caveolae rather than to the lack of changes in other cell types.

Recent studies have renewed interest in caveolae as putative mechanoprotective organelles that disassemble in response to mechanical distension and that act as a membrane reservoir that protects cells from membrane rupture (Lo et al. 2015; Cheng et al. 2015). The model of partial bladder outlet obstruction used here is relevant in this regard as it entails remarkable distension-induced growth and organ remodeling. Bladder outlet obstruction for 7 days causes a three- to eight-fold increase in bladder weight, hypertrophy of the detrusor muscle layer and wide-reaching transcriptomic and proteomic changes (Krawczyk et al. 2016; Ekman et al. 2013, 2014). Because the bladder grows to cope with increased mechanical distension, outlet obstruction should test whether caveolae are important for chronic mechanical adaptation. Our analyses do not support this view. We found bladder growth to be very similar in WT and KO mice, as is STAT3 phosphorylation and the loss of contractile markers. The only subtle difference is a curtailed induction of the Golgi protein GM130. We have not determined the mechanistic basis of this change but one possibility is that caveolin-1, whose level is better maintained than that of cavin-1, is partly redistributed to the Golgi apparatus. The presence of caveolin-1 in this membrane might influence the cleavage of transcription factors such as Creb3l2, an ER/Golgi-associated transcription factor that controls GM130 (Krawczyk et al. 2016) but this possibility remains to be tested.

To summarize, we identified cavin-3 as a smooth-muscle-specific cavin that contributes to the formation of caveolae in vivo. The lack of cavin-3 destabilizes cavin-1 and caveolin-1 and alters the relative contribution of NO and endothelial-dependent hyperpolarization to relaxation by muscarinic receptor activation. This occurs in a setting of maintained arterial blood pressure. The lack of cavin-3 moreover reduces the density of caveolae in the urinary bladder but this is without effect on contractility or mechanically stimulated growth and protein synthesis.
